# Pathophysiology, echocardiographic evaluation, biomarker findings, and prognostic implications of septic cardiomyopathy: a review of the literature

**DOI:** 10.1186/s13054-018-2043-8

**Published:** 2018-05-04

**Authors:** Robert R. Ehrman, Ashley N. Sullivan, Mark J. Favot, Robert L. Sherwin, Christian A. Reynolds, Aiden Abidov, Phillip D. Levy

**Affiliations:** 1 Department of Emergency Medicine, Wayne State University School of Medicine, Detroit Medical Center/Sinai-Grace Hospital, 4201 St. Antoine, Suite 3R, Detroit, MI 48201 USA; 2 Department of Emergency Medicine, Wayne State University School of Medicine, St. John Hospital and Medical Center, 22101 Moross Rd, Detroit, MI 48236 USA; 3 Department of Emergency Medicine, Cardiovascular Research Institute, Wayne State University School of Medicine, 540 E. Canfield, Detroit, MI 48201 USA; 4 Division of Cardiology, Wayne State University School of Medicine, John D. Dingell VA Medical Center, 3990 John R. 4 Hudson, Detroit, MI 48377 USA; 5 Department of Emergency Medicine, Wayne State University School of Medicine, Detroit Medical Center/Detroit Receiving Hospital, 4201 St. Antoine, Suite 3R, Detroit, MI 48201 USA

**Keywords:** Echocardiography, Sepsis, Troponin, B-type natriuretic peptide, Ultrasound

## Abstract

**Background:**

Sepsis is a common condition encountered by emergency and critical care physicians, with significant costs, both economic and human. Myocardial dysfunction in sepsis is a well-recognized but poorly understood phenomenon. There is an extensive body of literature on this subject, yet results are conflicting and no objective definition of septic cardiomyopathy exists, representing a critical knowledge gap.

**Objectives:**

In this article, we review the pathophysiology of septic cardiomyopathy, covering the effects of key inflammatory mediators on both the heart and the peripheral vasculature, highlighting the interconnectedness of these two systems. We focus on the extant literature on echocardiographic and laboratory assessment of the heart in sepsis, highlighting gaps therein and suggesting avenues for future research. Implications for treatment are briefly discussed.

**Conclusions:**

As a result of conflicting data, echocardiographic measures of left ventricular (systolic or diastolic) or right ventricular function cannot currently provide reliable prognostic information in patients with sepsis. Natriuretic peptides and cardiac troponins are of similarly unclear utility. Heterogeneous classification of illness, treatment variability, and lack of formal diagnostic criteria for septic cardiomyopathy contribute to the conflicting results. Development of formal diagnostic criteria, and use thereof in future studies, may help elucidate the link between cardiac performance and outcomes in patients with sepsis.

## Background

Septic cardiomyopathy (SC) is often diagnosed when some acute perturbation in cardiac function exists in the setting of sepsis. At present, no formalized or consensus definition of SC exists, representing a critical knowledge gap. Complexity of the cardiovascular system, myriad methods of assessment, and variations in the pre-septic state of the heart make elucidation of a cause-and-effect relationship difficult.

SC has been recognized for 40 years [1, 2] and may be present in up to 44% of patients [3, 4], but it remains incompletely understood. While results are varied, some studies suggest that mortality is two to three times greater when SC is present [5, 6]. Understanding how the heart behaves is critical when making treatment decisions for septic patients. For example, aggressive fluid resuscitation has been integral in the treatment of sepsis for nearly two decades, but recent literature suggests that excessive fluid resuscitation is deleterious in some patients [7, 8]. Variations in myocardial performance could explain, at least in part, these observed differences.

Early studies utilized invasive assessment methods or radionuclide imaging [4, 9]; while providing a plethora of data, these techniques are of limited utility to emergency and critical care physicians given that they cannot be performed at the point-of-care and are difficult to repeat.

Echocardiography, however, is widely available, non-invasive, and easily repeatable, making it an optimal modality for evaluation of SC. Measurement of serum cardiac biomarkers provides separate, but related, information about the state of the heart [10] and thus may be complementary to echocardiographically derived data.

The purpose of this article is to review the extant literature on SC, with a focus on the evaluation and prognostic implications of various echocardiographic and laboratory measures thereof.

## Pathophysiology

The pathophysiologic cascade of sepsis begins when the host immune system responds to an invading pathogen, resulting in activation of the innate immune response [11]. This culminates in the generation and release of pro-inflammatory mediators and signaling molecules that may be physiologic (beneficial) or pathologic (harmful) to the host; concomitant release of anti-inflammatory mediators occurs as well. These molecules, acting through varied signal transduction pathways, which in some cases alter gene expression, activate both positive and negative feedback loops within the immune system [12]. Recent advances in oxidative lipidomics have identified products of upstream lipid metabolism that are involved in the initiation (eicosanoids) and recovery phases (lipoxins, resolvins) [13]. Sepsis-induced dysregulation of the normal immune response can lead to a variety of deleterious effects, including SC, multi-system organ failure, and ultimately death in some patients [5] .

Septic shock is often classified as a type of “distributive” shock—relative hypovolemia resulting from maldistribution of circulating volume due to peripheral vasodilation, glycocalyx dysfunction, and increased capillary permeability. It has also been described as a biphasic disorder with an early, hyperdynamic phase (high cardiac output (CO), low systemic vascular resistance (SVR), warm extremities) and a late, hypodynamic phase (low CO, poor perfusion) [5, 6]. Circulating inflammatory mediators are believed to be the causative agents, acting directly on cardiomyocytes and the peripheral vasculature, which affects myocardial performance via alterations in SVR and venous return.

While preload augmentation has long been a primary intervention for sepsis and can increase CO via the Frank-Starling mechanism, its ability to do so depends on the functional state of the heart. However, measures of preload, such as central venous pressure (CVP) and inferior vena cava (IVC) dimensions, provide limited information on underlying cardiac function. Even when volume responsiveness is suggested by low CVP, increased respiratory variation of IVC diameter, or other invasive methods, guidance of resuscitation based on these measures has not been found to improve outcomes [14–16].

Changes in afterload also affect the ability of the heart to deliver blood to the peripheral tissues. Thus, a heart with poor intrinsic contractility may be able to increase CO when SVR is low—thereby giving the impression of normal function—when in fact systolic performance is impaired. This dysfunction may only become apparent when SVR returns to normal via natural (recovery from sepsis) or artificial means (vasopressor use). Boissier et al. [17] demonstrated such an inverse relationship between ejection fraction and SVR in septic patients.

**Fig. 1:**
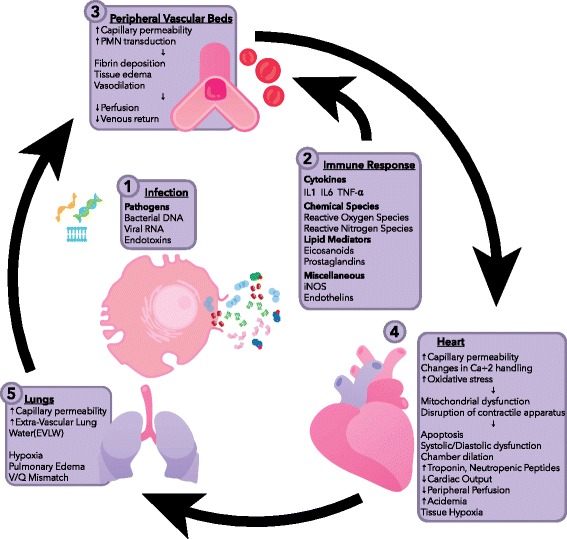
Pathophysiology of septic myocardial dysfunction. IL interleukin, iNOS induced nitric oxide synthase, *PMN* polymorphonuclear cell, *TNF* tumor necrosis factor

An early hypothesis, based on animal models, was that SC was caused by global myocardial ischemia resulting from decreased coronary blood flow [18]. Subsequent investigation, including human studies, showed preserved or increased coronary perfusion in some septic patients [19]. Derangements in cardiomyocyte physiology are still believed to play a role, but at the microcirculatory, rather than the macrocirculatory, level [20]. Inflammatory molecules are thought to be responsible for this via pleiotropic effects [5]. Damage occurs via changes in endothelial permeability, leading to edema, increased neutrophil transduction into the interstitium, fibrin deposition, and in some cases, activation of the coagulation cascade [21]. Increased oxidative stress may induce mitochondrial dysfunction and disruption of normal calcium handling [22]—critical events given the high-energy demands of cardiac tissue. The exact mechanism that culminates in cardiac dysfunction is not clear; one theory posits that myocardial edema leads to disruption or malfunction of the contractile apparatus [23]. Autonomic dysregulation leading to decreased expression of adrenergic receptors and thus resistance to endogenous catecholamines may also be present [22]. Figure 1 illustrates the complex interactions between host and pathogen factors that contribute to the development of SC.

Despite such pathology, a cardinal feature of SC is its apparent reversibility, with many studies reporting that patients’ cardiac function recovered fully to their pre-morbid state [1, 5, 20, 24]. Cardiac magnetic resonance has detected changes suggesting myocardial edema or an altered metabolic state, a pattern distinct from that seen with ischemia and necrosis, the former being consistent with reversibility [25]. Thus, some theorize that SC represents a protective “hibernating” state [26], as has been demonstrated in subsets of patients with acute myocardial ischemia [27]. A reversible—and incompletely understood—takotsubo pattern has also been described in septic patients. The physiologic stresses of sepsis are thought to play a role, but its place within the SC continuum is not clear given the paucity of extant data [28].

### Echocardiography: diagnosis and prognosis

A summary of the echocardiographic parameters that have been used in the evaluation of SC, and their limitations, is listed in Table 1. Table 2 summarizes the literature on echocardiography in SC discussed in the ensuing sections. According to GRADE guidelines [29], the quality of evidence on this topic is “low” or “very low”. A summary of the major limitations within the literature on septic cardiomyopathy is provided in Table 3.

**Table 1:** Summary of echocardiographic variables that have been used to evaluate septic cardiomyopathy

	Echo parameter	Measurement	Significant findings	Strengths	Limitations	Comments
Left ventricle						
Systolic	EF	EF = (LVEDV − LVESV)/LVEDV × 100	Abnormal LV systolic function suggested by EF < 52% in men or < 54% in women [63]	Frequently used; easy to acquire	Biplane Simpson’s Method of Discs is the only currently recognized method per ASE guidelines. Requires adequate image quality; EF will vary with beat to beat variation; highly dependent on loading conditions of the LV	Familiar to all clinicians. Fails to accurately identify all patients with SC
	GLS	GLS (%) = (MLs − MLd)/MLd	Abnormal LV systolic function suggested by peak GLS < − 20% [63]	Improved prognostication over LVEF for determining LV systolic function; independent of angle	Requires adequate image quality, absence of foreshortening, and three standard apical views. No consensus on abnormal values due to variability in vendors and analytic software	Speckle-tracking technology increasingly available
	Systolic mitral annular velocity (S′)	AVG peak systolic velocity (cm/s) of the mitral annulus is measured using spectral TDI	No current consensus recommendations on abnormal values; abnormal LV systolic function suggested by decreased S′ < 10 cm/s	Easy to acquire; less dependent on preload	Not well validated; heterogeneity of values depending on patient age; may be affected by regional wall motion abnormalities, annular calcifications, and prosthetic valves	Able to be performed with old or modern equipment
	MAPSE	MAX systolic plane excursion of the lateral mitral annulus (cm) is measured using M-mode	No current consensus recommendations on abnormal values; abnormal LV systolic function suggested by decreased MAPSE < 1 cm	Easy to acquire	Requires adequate M-mode cross section; may be affected by regional wall motion abnormalities, annular calcifications, and prosthetic valves	Conceptually similar to GLS, but able to be performed without speckle-tracking or Doppler
	MPI	MPI = (TST − ET)/ET	Abnormal LV function suggested by MPI > 0.40 [78]	Derived from simple time interval recordings; less load dependency; does not rely on geometric assumptions	Limited validated research; both systolic and diastolic dysfunction can result in an abnormal MPI; requires accurate measurements of cardiac time intervals	Most clinicians have little familiarity with this measure
Diastolic^a^	e’	Peak e’ velocity (cm/s) in early diastole measured using PW Doppler at lateral and septal basal regions and then averaged	Abnormal LV diastolic function suggested by e’ (septum) < 7 cm/s or e’ (lateral) < 10 cm/s [51]	Easy to acquire; less dependent on preload	Regional wall motion abnormalities and CAD will affect the measurement; heterogeneity of values depending on patient age	Highly studied and shows promise as a simplified method of assessing diastolic function in SC
	E/e’	E/e’ = E (cm/s)/*e*’ (cm/s)	Abnormal LV diastolic function suggested by average E/e’ > 14 cm/s [51]	Easy to acquire	Regional wall motion abnormalities and CAD will affect the measurement; limited accuracy in normal patients, or patients with annular calcification, mitral valve or pericardial disease; heterogeneity of values depending on patient age	Similar potential as e’; allows estimation of pulmonary capillary wedge pressure via formula: E/e’ + 4.6
Right ventricle						
Systolic	TAPSE	MAX plane of systolic excursion of the lateral tricuspid annulus (mm) is measured using M-mode	Abnormal RV systolic function suggested by TAPSE < 17 mm [63].	Easy to acquire; well demonstrated prognostic value	Only evaluates longitudinal myocardial shortening; dependent on angle; may be affected by LV systolic dysfunction and significant tricuspid regurgitation	Important associations with mortality [64]

*ASE* American Society of Echocardiography, *AVG* average, *CAD* coronary artery disease, *EF* ejection fraction, *ET* ejection time, *GLS* global longitudinal strain, *LVEDV* left ventricular end diastolic volume, *LVEF* left ventricular ejection fraction, *LVESV* left ventricular end systolic volume, *MAPSE* mitral annular plane of systolic excursion, *MAX* maximum, *MLd* myocardial length end-diastole, *MLs* myocardial length end-systole, *MPI* myocardial performance index, *PW* pulsed wave, *TAPSE* tricuspid annular plane of systolic excursion, *TDI* tissue Doppler imaging, *TST* total systolic time

^a^ The table provides general guidelines for annular velocities and ratios based on the 2016 recommendations for the evaluation of left ventricular diastolic function. Based on these recommendations, there are four recommended variables and abnormal values for determining diastolic dysfunction: septal e’ < 7 cm/s, lateral e’ < 10 cm/s, average E/e’ ratio > 14, LA volume index > 34 mL/m^2^, and peak TR velocity > 2.8 m/s. Left ventricular diastolic function is considered abnormal if more than half of these variables exceed their cutoff values. The 2009 recommendations for the evaluation of left ventricular diastolic function previously suggested an abnormal septal e’ < 8 cm/s and lateral e’ < 10 cm/s. Existing studies have relied upon a simplified version of the 2009 ASE guidelines for assessing diastolic function (using only e’ and E/e’); while one study [60] found that measures beyond e’ and E/e’ provided limited additional prognostic information, whether application of the updated guidelines would improve the prognostic utility of diastolic assessment requires further study

**Table 2:** Summary of selected articles on septic cardiomyopathy

	Echo parameter	Study	Study design/setting	*N*	Measured outcome	Results
Left ventricle						
Systolic	EF	Sevilla Berrios et al. (2014) [33]	Meta-analysis	585	To evaluate the significance of reduced LVEF in patients with severe sepsis and septic shock. Primary outcome was association between depressed LVEF and 30-day mortality	Depressed LVEF had a sensitivity of 52% (95% CI 29–73%) and specificity of 63% (95% CI 53–71%) for mortality and was therefore not a sensitive nor specific predictor of mortality
		Huang et al. (2013) [32]	Meta-analysis	762	To evaluate the association of both reduced LVEF and increased LV dimensions with mortality in patients with severe sepsis and septic shock	No significant difference in LVEF and LV dimensions in survivors vs non-survivors
		Jardin et al. (1999) [24]	Single-center prospective cohort study	90	To evaluate changes in LV function, including LVEF and LV volumes, during volume resuscitation in patients with septic shock	LVEF was depressed in all patients. LV parameters were additionally unaffected by fluid loading
		Parker et al. (1984) [1]	Single-center prospective cohort study	20	To evaluate cardiac function in septic shock	10/20 patients (50%) had depressed LVEF (< 0.40). Mean LVEF was lower among survivors (LVEF 0.32 ± 0.04) when compared to non-survivors. Mean ESV and EDV were increased in survivors
	GLS	Boissier et al. (2017) [17]	Single-center prospective cohort study/ICU	132	To evaluate the role of GLS, LVEF, and TDI in patients with septic shock. Primary outcome was the role of loading conditions on evaluation of cardiac contractility	GLS was impaired in a majority of the patients (> 70%); however, feasibility was limited (< 50%)
		Chang et al. (2015) [39]	Multi-center prospective cohort study/ICU	111	To evaluate LV function, as well as the prognostic value of GLS, in septic patients. Primary outcome was both ICU and hospital mortality	GLS is an independent prognostic indicator of ICU mortality. Patients with GLS ≥ − 13% had higher ICU mortality rates (HR 4.34; *p* < 0.001)
		De Geer et al. (2015) [43]	Single-center prospective cohort study/ICU	50	To evaluate GLS in patients with septic shock. Primary outcomes were mortality at 30 and 90 days	GLPS did not correlate between survivors and non-survivors and therefore could not be used to predict mortality
		Innocenti et al. (2016) [41]	Single-center prospective cohort study/ED observation unit	147	To evaluate LVEF and GLS in septic patients. Primary outcome was all-cause mortality at 7 days	LVEF is not an independent indicator of prognosis
		Kalam et al. (2014) [37]	Meta-analysis	5721	To assess if GLS is a more accurate predictor of cardiovascular outcome compared to LVEF. Primary outcome was all-cause mortality. Secondary outcome was composite endpoint including cardiac death, malignant arrhythmia, and hospitalization	GLS is a better predictor of adverse outcomes (HR 0.50; *p* < 0.002) and mortality (HR 1.62; *p* = 0.009) than LVEF (HR 0.81; *p* = 0.572)
		Ng et al. (2016) [38]	Case–control study/ICU	62	To evaluate the role of GLS in the diagnosis of SMD. Primary outcome was to compare GLS values in patients with septic shock compared to patients with only sepsis	There was a significant difference in GLS values (− 14.5 vs –18.3%, *p* < 0.001) between patients with septic shock and sepsis. LVEF was not statistically significant between patients with septic shock and patients with sepsis
		Orde et al. (2014) [42]	Single-center prospective cohort study/ICU	60	To evaluate GLS in patients with severe sepsis or septic shock. Primary outcomes were mortality at 30 days and 6 months	No difference in mortality for LV GLS or GLS rate in survivors compared with non-survivors at 30 days or 6 months
		Palmieri et al. (2015) [40]	Single-center prospective cohort study/ED observation unit	115	To evaluate LV EF and peak GLS in patients with sepsis and septic shock. Primary outcome was death by any cause at 28 days from hospitalization	Abnormal GLS correlates significantly with mortality rate at 28 days. GLS values close to 0 demonstrated a higher mortality (HR 1.16%; *p* = 0.05).
		Zaky et al. (2016) [44]	Single-center prospective cohort study/ICU	54	To evaluate LVLS in patients with sepsis or septic shock. Primary outcomes were mechanical ventilation, ICU and hospital length of stay, and in-hospital mortality	Global LVLS was not associated with rates of mechanical ventilation, ICU or hospital length of stay, or in-hospital mortality
	Systolic mitral annular velocity (S′)	Chang et al. (2015) [39]	Multi-center prospective cohort study/ICU	111	To evaluate LV function, as well as the prognostic value of GLS, in septic patients. Primary outcome was both ICU and hospital mortality	There was no statistically significant difference in S′ between ICU non-survivors compared to survivors (11.0 ± 4.3 vs 11.4 ± 4.0; *p* < 0.66)
		Weng et al. (2012) [49]	Single-center prospective cohort study/ICU	61	To evaluate the prognostic significance of several TDI variables, including systolic mitral annular velocity, S′, in patients with septic shock. Primary outcome was all-cause mortality	Non-survivors had a higher S′ when compared to survivors (11.0 vs 7.8 cm/s; *p* < 0.0001). Patients with S′ > 9 cm/s had a higher mortality rate (75 versus 17%; *p* < 0.0001). S′ > 9 cm/s had SN 75% and SP 86% to predict 90-day mortality
		Weng et al. (2013) [50]	Single-center prospective cohort study/ICU	51	To evaluate LV longitudinal systolic dysfunction and LV intraventricular systolic asynchrony assessed by TDI in patients with septic shock and normal LVEF. Primary outcome was all-cause mortality at 28 days	Normal EF, LV longitudinal systolic dysfunction and LV systolic asynchrony assessed by TDI within 24 h of onset of septic shock were associated with improved mortality at 28 days
	MAPSE	Zhang et al. (2017) [65]	Case-control study/ICU	45	To evaluate LVEF, MAPSE, Sa, and TAPSE in patients with septic shock. Primary outcome was sepsis	MAPSE values were significantly lower in septic patients when compared to non-septic patients (*p* ≤ 0.001)
	MPI	Nizamuddin et al. (2017) [78]	Single-center prospective cohort study/ICU	47	To assess if changes in LV MPI were associated with higher 90-day mortality in patients with severe sepsis. Primary outcome was all-cause mortality	Decline in MPI over the initial 24-h study period was associated with higher mortality at 90 days (*p* = 0.04)
Diastolic	e’ and E/e’	Brown et al. (2012) [52]	Single-center prospective cohort study/ICU	78	To evaluate whether severity of diastolic dysfunction predicts mortality in patients with severe sepsis or septic shock. Primary outcome was mortality at 28 days	Grade I diastolic dysfunction was associated with increased mortality; grades II/III were not associated with increased mortality
		Landesberg et al. (2012) [54]	Single-center prospective cohort study/ICU	262	To evaluate the association between diastolic dysfunction and mortality in severe sepsis and septic shock. Primary outcomes were in-hospital mortality and overall mortality at 6 months to 2 years	Decreased septal e’ or increased septal E/e’ were the strongest independent predictors of mortality (HR 0.76, *p* ≤ 0.001 and HR 1.08, *p* ≤ 0.001, respectively)
		Rolando et al. (2015) [57]	Single-center prospective cohort study/ICU	53	To evaluate the prognostic significance of myocardial dysfunction, including E/e’ ratio, in patients with severe sepsis and septic shock. Primary outcome was hospital mortality	E/e’ is an independent predictor of hospitality mortality (OR = 1.36; *p* = 0.02). An E/e’ > 11 had a sensitivity of 50% and specificity of 94% for predicting ICU mortality
		Sanfilippo et al. (2017) [59]	Meta-analysis	1507	To evaluate the association of e’ and E/e’ with mortality in patients with severe sepsis or septic shock	A significant association was found between mortality and both a lower e’ (SC 0.33; 95% CI 0.05, 0.62; *p* = 0.02) and higher E/e’ (SC 0.33; 95% CI – 0.57, − 0.10; *p* = 0.006) in patients with severe sepsis and/or septic shock. There was high overall heterogeneity in both e’ and E/e’ analysis
		Sturgess et al. (2010) [56]	Single-center prospective cohort study/ICU	21	To evaluate the prognostic significance of TDI and cardiac biomarkers in septic shock. Primary outcome was hospital mortality	E/e’ is an independent predictor of hospital survival and is a better prognosticator than cardiac biomarkers. E/e’ was greater in non-survivors than survivors (15.32 ± 2.74 vs 9.05 ± 2.75, respectively; *p* = 0.0002)
		Lanspa et al. (2016) [60]	Single-center prospective cohort study/ICU	167	To compare the feasibility and prognostic significance of a simplified definition of diastolic dysfunction (using e’ and E/e’) with 2009 ASE guidelines. Primary outcome was 28-day mortality	Simplified definition had better feasibility (87 vs 35%); similar clinical outcomes between groups suggesting limited utility of LAVI and DT in this setting
Right ventricle						
Systolic	TAPSE	Gajanana et al. (2015) [64]	Single-center prospective cohort study/ICU	120	To evaluate the prognostic value of TAPSE in patients with critical illness	A reduced TAPSE measurement (< 2.4 cm) was correlated with increased in-hospital mortality (χ(2) = 4.6, *P* = 0.03) and a longer length of hospital stay
	TAPSE TDI RV FAC	Vallabhajosyula et al. (2017) [67]	Single-center retrospective cohort study/ICU	388	To evaluate the prognostic significance RV dysfunction in patients with severe sepsis and septic shock. Primary outcome was 1-year survival	Isolated RV dysfunction is an independent predictor of 1-year survival (HR 1.6; *p* = 0.002). Combined RV/LV dysfunction was not an independent predictor of 1-year survival (HR 0.9; *p* = 0.52)

*ASE* American Society of Echocardiography, *CI* confidence interval, *DT* mitral inflow deceleration time, *EDV* end diastolic volume, *ESV* end systolic volume, *FAC* fractional area change, *GLPS* global longitudinal peak strain, *GLS* global longitudinal strain, *HR* hazard ratio, *ICU* intensive care unit, *LAVI* left atrial volume index, *LV* left ventricle, *LVEF* left ventricular ejection fraction, *LVLS* left ventricular longitudinal strain, *MAPSE* mitral annular plane systolic excursion, *OR* odds ratio, *RV* right ventricle, *Sa* tissue Doppler velocity measurement of mitral annulus, *SC* septic cardiomyopathy, *SMD* standard mean difference, *SN* sensitivity, *SP* specificity, *STE* speckle tracking echocardiography, *TDI* tissue Doppler imaging

**Table 3:** Gaps and general limitations of the septic cardiomyopathy literature

	Limitations	Potential Impact
Patient-related factors	Observational study designs with generally small sample sizes	High degree of confounding and bias; elucidation of true causal relationships not possible
	Heterogeneous sepsis classification (SOFA, SIRS) and severity	Difficult to make conclusions across varied populations; prognostic value of echocardiography findings confounded by collinearity between severity of disease and adverse outcomes
	Pre-septic cardiac function largely unknown	Acute versus chronic dysfunction may portend different prognosis
	Variation in co-morbidities	Complex interaction between pre-existing illnesses, acute infection, and treatment renders cross-patient comparisons difficult
	Variation in treatments (mechanical ventilation, vasopressors, inotropes)	Therapeutic interventions likely affect cardiac performance and echocardiographic measurements and may alter outcomes
Echocardiography-related factors	Variable timing of initial echocardiogram	Normal progression of disease (natural history) and treatment prior to initial exam may alter findings
	Variability of timing and number of repeat echocardiograms	Ongoing resuscitation may alter cardiac performance via intrinsic (e.g., increased contractility) or extrinsic (change in loading conditions) factors
	Reference ranges derived in stable patients	Unknown how/if normal values are applicable in the setting of sepsis
	GLS values not standardized across ultrasound vendors	Difficult to compare GLS values across US systems

*GLS* global longitudinal strain, *SIRS* systemic inflammatory response syndrome, *SOFA* Sequential Organ Failure Assessment, *US* ultrasound

### Left ventricle: systolic function

#### Ejection fraction

Systolic dysfunction, as measured by left ventricular ejection fraction (LVEF), was one of the first described parameters to assess for SC. Parker et al., in 1984 [1], reported that approximately 50% of their patients with septic shock had reduced LVEF. Counter-intuitively, they found low mean LVEF amongst survivors compared to non-survivors. Jardin et al. confirmed these findings [24] and further reported that LV parameters were unaffected by fluid-loading in non-survivors. Unfortunately, several follow-up studies found no difference in LVEF between survivors and non-survivors of septic shock [30, 31]. Two recent meta-analyses that included 1247 patients failed to find any meaningful relationship between LV parameters and mortality in septic shock [32, 33].

#### Global longitudinal strain

Strain imaging is a novel technique based on regional myocardial deformation. The most frequently used strain parameter is global longitudinal strain (GLS), which represents the mean longitudinal strain value from each segment of the LV [34].

**Fig. 2:**
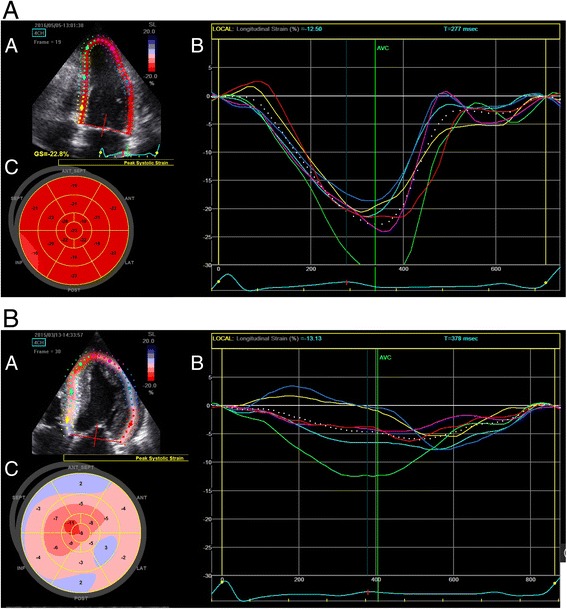
**a** Speckle-tracking analysis of a patient with normal systolic left ventricular (LV) function. 2D image showing speckles within the LV being tracked by the ultrasound Machine Software (*A*). Graphical representation of movement of speckles throughout the cardiac cycle (x-axis, longitudinal strain; y-axis, time in msec), with each *line* representing a different segment of the LV; large negative values represent movement of speckles towards one another during contraction representing normal function (*B*). Bullseye map showing global longitudinal strain values throughout the LV (*C*). **b** Speckle-tracking analysis of a patient with severely reduced left ventricular (LV) systolic function. A 2D image showing speckles within the LV being tracked by the ultrasound machine software (*A*). Graphical representation of movement of speckles throughout the cardiac cycle (x-axis, longitudinal strain; y-axis, time in msec) with each *line* representing a different segment of the LV; note smaller negative values with variable time to peak strain representing reduced LV function with mechanical dyssynchrony (*B*). Bullseye map showing global longitudinal strain values throughout the LV; *blue zones* represent areas of the LV where there is lengthening of the segments during systole rather than shortening (*C*)

Most commonly, GLS is calculated using speckle-tracking echocardiography (STE) [35]. STE is a semi-automated, post-processing computer algorithm that tracks user-selected regions of the myocardium (“speckles”) during the cardiac cycle. As fibers contract during systole, speckles move closer together, represented by negative values. Larger negative values represent greater deformation in systole and thus improved LV function (Fig. 2).

Strain imaging has the ability to detect subtle changes in LV systolic function prior to decline in LVEF [36]. A meta-analysis by Kalam et al. found that GLS was a better predictor of adverse outcomes, including mortality, than LVEF patients with heart failure and other cardiac diseases [37]. Ng et al. [38] found that patients with septic shock had more LV dysfunction, as measured by GLS, than matched controls with sepsis but without shock. Chang et al. [39] prospectively enrolled 111 ICU patients with septic shock. LVEF was similar between in-hospital survivors and non-survivors, but GLS was significantly better in survivors compared to non-survivors, with an even greater difference for ICU mortality [39]. Palmieri et al. [40] reported similar findings. When dichotomized to “normal” (< − 14%) or “abnormal”, Innocenti et al. [41] reported lower mortality at 7 and 28 days for those with normalized GLS.

Conversely, Orde et al. [42] found that although a greater number of patients had LV dysfunction identified by STE than LVEF (69 versus 33%), there was no difference in GLS between survivors and non-survivors at 30 days or 6 months; De Geer et al. [43] also found no difference or improvement in GLS between survivors and non-survivors at 30 and 90 days. Finally, Zaky et al. [44] reported similar strain values in survivors and non-survivors; however, apical segments were excluded from their strain analysis, making interpretation of these results somewhat difficult. In terms of feasibility of performing STE in septic patients, studies have reported exclusion rates for poor image quality from 1.5–20% [40–43, 45, 46]. De Geer et al. [46] found GLS to be the most reproducible measure for assessing cardiac function in patients with septic shock, with intraclass correlation coefficients for inter- and intraobserver variability of 0.91 (0.74–0.95, *p* < 0.001), and 0.89 (0.55–0.97, *p* = 0.002), respectively.

#### Other considerations

Systolic function can also be assessed using tissue Doppler imaging (TDI)-derived velocity of the mitral annulus or by mitral annular plane systolic excursion (MAPSE). Peak systolic velocity of the mitral annulus, denoted S′, has been shown to have prognostic value in a variety of cardiovascular illnesses [47] and is relatively afterload-independent [17]. MAPSE is the linear distance the mitral annulus moves towards the LV apex during systole and has demonstrated similar utility [48].

The evidence with regard to the utility of these variables in septic patients is conflicting. Weng et al. [49] found a linear correlation between S′ and LVEF in septic shock, and that non-survivors had higher S′ than survivors; Boissier et al. [17] reported similar findings. A study of septic patients with LVEF > 50% also noted lower S′ in survivors compared to non-survivors [50]. Conversely, Chang et al. [39] found no association between S′ and ICU or in-hospital mortality.

### Left ventricle: diastolic function

In the absence of infiltrative or restrictive cardiac disease, diastole is the main determinant of LV compliance [51]. While initially low in SC, regardless of systolic function, as diastolic function worsens, left ventricular end-diastolic pressure (LVEDP) can rise. Preload augmentation, via increased left ventricular end-diastolic volume, can lead to further elevation in LVEDP—Brown et al. [52] found that diastolic dysfunction (DD) was more common in patients administered larger volumes of IVF. Mahjoub et al. [53] reported less increase in LVEDP in volume-responsive septic patients than in non-responders, demonstrating the varied effects of IVF-loading, depending on underlying cardiac function. A putative explanation of the detrimental effects of DD in sepsis is that elevated left-sided pressure increases pressure in the pulmonary circulation, the right heart, and the peripheral tissues, which leads to increases in extravascular lung water (EVLW) and tissue edema.

The preferred method for evaluation of diastolic function is TDI-derived velocity of the mitral annuls during early diastole (*e*’), with lower values corresponding to worse diastolic function [51]. The ratio of the peak trans-mitral inflow velocity in early diastole (*E*) to early diastolic mitral annular velocity (*E*/*e*’) has been found to correlate with left-atrial pressure—a surrogate for pulmonary capillary wedge pressure [51]; higher values reflect increased pressure.

Prognostic ability of *E*/*e*’ and *e*’ have been evaluated in septic patients, with conflicting results. Three studies reported lower e’ in non-survivors compared to survivors [54–56]; two others found that elevated E/e’ was an independent predictor of in-hospital mortality [56, 57]. A 2015 meta-analysis [58] of 636 patients found a prevalence of DD of 20–57% and a relative risk of death of 1.82 (1.12–2.97, *p* = 0.002; I^2^ = 77%). A 2017 update [59], of 1507 patients, found lower lateral e’ and higher E/e’ amongst non-survivors. Interestingly, Brown et al. [52] found increased mortality only for patients with low-grade DD (impaired relaxation with minimally elevated LVEDP); however, these patients received significantly less fluid than those with more severe DD—2.6 versus 5.5 l—prior to the initial echocardiogram and thus they may have been inadequately resuscitated. While e’ is considered to be relatively preload independent [51], this latter finding suggests there is a link between fluid loading and derangement in diastolic function. In contrast, several other studies failed to detect an association between DD and mortality (early or late—up to 1 year) in severe sepsis and septic shock [30, 31, 39, 43, 49].

Lanspa et al. [60] found better feasibility of a simplified diastolic evaluation compared to the 2009 American Society for Echocardiography (ASE) guidelines, without loss of predictive value (see Tables 1 and 2 for further details).

### Right ventricle

Dysfunction of the right ventricle (RV) contributes to morbidity and mortality in a variety of conditions, including heart failure and pulmonary hypertension [61, 62]. Tricuspid annular plane systolic excursion (TAPSE) is the easiest and most reproducible measure of RV function [63]. Reduced TAPSE has been correlated with increased mortality in critical illness [64] and detected in septic ICU patients when compared to non-septic ICU controls [38, 65]. Whether RV dysfunction is a manifestation of disease severity—and thus associated with poor outcomes—or a causative factor of morbidity and mortality is unknown. Contributors to RV dysfunction include LV dysfunction, hypoxia, hypercarbia, mechanical ventilation with high positive end-expiratory pressure, atelectasis, and fluid overload [42, 62]. Pulmonary hypertension induced (or exacerbated by) acute respiratory distress syndrome or pulmonary sources of sepsis can also contribute to RV dysfunction; isolated RV dysfunction may be more common in patients with these conditions compared to those without.

Reported prevalence of RV dysfunction in sepsis varies, from 31 to 83% [31, 57]. As with other echocardiographic variables, investigation of RV parameters has produced conflicting results. Traditional parameters of increased RV size and dysfunction have been associated with increased mortality in some studies [55, 66], but not others [31, 39, 54, 57]. A retrospective study of 388 septic ICU patients [67] found isolated RV dysfunction to be an independent predictor of 1-year mortality; combined RV/LV dysfunction showed no such relationship. A meta-analysis of 412 patients found no association between RV function and mortality [32]. Orde et al. [42] reported that reduced RV free wall strain (by STE) was associated with 6-month mortality (− 16.0 ± 5.7 versus − 19.3 ± 4.9, *p* ≤ 0.05); these results are promising but require validation.

### Biomarkers

Abnormal cardiac biomarkers, primarily troponin and natriuretic peptides (NPs), are another potential indicator of myocardial dysfunction that provide unique but related information about the heart [10]. Troponin elevation in the setting of sepsis may reflect altered cardiomyocyte permeability or necrosis from vascular injury rather than atherosclerotic disease [68], but determining whether it is related to SC or another condition (e.g., renal disease) is difficult.

Contemporary troponin assays (cTnI, cTnT) have been studied in septic patients, with concentrations generally rising with increasing disease severity; short-term non-survivors often have greater elevations than survivors [10, 69], but the results are not uniform [70]. Vallabhajosysula et al. [71] reported that, in septic ICU patients, elevated TnT on admission was associated with higher in-hospital and 1-year mortality compared to patients whose TnT was not elevated; no such associations were found for elevated ΔTnT. High-sensitivity troponin (hsTnT) has also been reported to rise with increasing severity of sepsis, but with unclear prognostic implications: Rosjo et al. [70] reported greater hsTnT elevation in non-survivors, but no independent association with mortality; Masson et al. [72] found that an elevated hsTnT on day 7 (but not day 1) and a > 20% rise from day 1 to day 2 were associated with increased mortality.

B-type NP (BNP), which is released in response to wall stress, reflects myocardial loading conditions and provides indirect functional information—as the heart moves towards the unfavorable portion of the Frank-Starling curve, wall stress, and BNP, rise [73, 74]. As with BNP, amino-terminal pro-BNP (NT-proBNP) may be elevated in sepsis, particularly with increasing disease severity, and is more likely to be elevated in non-survivors compared to survivors [69, 72–75]. In one study, NT-proBNP was a better predictor of 90-day mortality than hsTnT [72]. Concomitant study of biomarkers and echocardiographic variables has not produced reliable results. No associations have been found between LV systolic function and contemporary troponin assays [31, 44]; one study reported an inverse relationship between LVEF and hsTnT [54], another found a weak association between hsTnT and declining GLS (*r* = 0.35) [43], and a third found no relationship [55]. Dilation of the RV and LV DD have been variably associated, with direct correlations for cTnT, hsTnT, and NT-proBNP in some studies [54, 55, 76], but not in others [31]. Sturgess et al. [56] found that E/e’ > 15 was a better predictor of in-hospital mortality than TnT or NTproBNP.

Some of this variability may be due to the fact that cardiac biomarker concentrations above the reference range are common in sepsis, with prevalence as high as 84% for cTnT [77], 98% for NT-proBNP [75], and 100% for hsTnT [70]. Existing data are limited by residual confounding from clinical contributors, including age, disease severity, comorbidities (including flow-limiting CAD), and treatment. Pending future studies, existing information on cardiac biomarkers can be summarized by noting that abnormalities in the setting of sepsis are likely, but such abnormalities, in and of themselves, do not equate to a diagnosis of SC, nor do they provide clear, independent prognostic information.

## Implications for treatment

Currently, no treatment recommendations exist that specifically address the presence of SC. Patients with SC may be at greater risk for excessive fluid resuscitation and more likely to require inotropic support, as hypoperfusion would less likely be corrected by IVF administration alone. However, data comparing use of vasopressors, inotropes, and other treatments in patients with and without SC are scant and subject to extensive confounding. Where treatment data are stratified by presence or absence of SC [31, 44, 52, 78], only two found significant differences between groups. Brown et al. [52] found that patients with less severe DD received less IVF than those with more significant abnormalities (2.6 versus 5.5 l). Pulido et al. [31] reported higher doses of noradrenaline (norepinephrine) in patients with LV and RV systolic dysfunction, but no overall difference in the number of patients receiving it. Establishment of a standardized, objective definition of SC and adoption of more uniform study protocols amongst research groups would improve understanding of differences in treatment requirements for patients with SC.

Positive inotropic agents have the same putative benefits of vasopressors—increasing CO, thereby improving oxygen delivery to the peripheral tissues. Recommendations to titrate therapy to central venous oxygen saturation > 65% are of uncertain utility, as a normal value does not necessarily indicate adequate resuscitation [79]. Furthermore, excessive β stimulation may be harmful, and there is some evidence that β-blockade may be beneficial in some patients [80]. Preliminary trials of levosimendan, a calcium sensitizer and positive inotrope, reported reduced mortality [81], but no benefit was found in a subsequent larger study [82].

## Conclusions

SC is a multi-factorial process that involves complex interactions between host and pathogen factors, and a full understanding of the disease process remains elusive. Prognostic implications of echocardiographic and biomarker findings are precluded by conflicting data from extant literature. Formal diagnostic criteria for SC do not exist; development of these, and studies based thereon, should be priorities for future research.

## Abbreviations

ASE: American society of echocardiography
BNP: B-type natriuretic peptide
CAD: Coronary artery disease
CI: Confidence interval
CO: Cardiac output
cTnT/cTnI: Contemporary troponin T/I
CVP: Central venous pressure
DT: Mitral inflow deceleration time
EDV: End diastolic volume
ESV: End systolic volume
GLPS: Global longitudinal peak strain
GLS: Global longitudinal strain
HR: Hazard ratio
hsTnT: High-sensitivity troponin T
ICU: Intensive care unit
IVC: Inferior vena cava
LAVI: Left atrial volume index
LV: Left ventricle
LVEF: Left ventricular ejection fraction
MAPSE: Mitral annular plane systolic excursion
msec: Millisecond
NP: Natriuretic peptide
NT pro-BNP: Amino-terminal pro-BNP
OR: Odds ratio
Sa: Tissue doppler velocity measurement of mitral annulus
SC: Septic cardiomyopathy
SN: Sensitivity
SP: Specificity
STE: Speckle tracking echocardiography
SVR: Systemic vascular resistance
TDI: Tissue doppler imaging
